# Conditions for and potential solutions associated with continuity of care for patients with complex care needs across Swedish regions with differing population densities

**DOI:** 10.1186/s12913-025-12649-1

**Published:** 2025-04-28

**Authors:** Linda Ljungholm, Charlotte Klinga, Mirjam Ekstedt, Anette Edin-Liljegren, Elin-Sofie Forsgärde

**Affiliations:** 1 Department of Health and Caring Sciences, Linnaeus University, Kalmar, Växjö, SE-391 82 Sweden; 2 Academic Primary Healthcare Centre, Stockholm Health Care Services, Stockholm, Sweden; 3 Department of Learning, Informatics, Management and Ethics, Medical Management Centre, Karolinska Institutet, Stockholm, SE-171 77 Sweden; 4 Department of Epidemiology and Global Health, Umeå University, Umeå, 901 87 Sweden; 5 The Centre for Rural Health, Region Västerbotten, Stationsgatan 3, Storuman, SE-923 31 Sweden

**Keywords:** Collaboration, Coordination, Communication, Continuity of patient care, Integrated care, Person-centered care, Patient care management

## Abstract

**Background:**

This study, conducted across three distinct geographical regions in Sweden, highlights the diverse conditions and challenges in healthcare provision. The study focuses on the sparsely populated northern regions of Sweden, the capital city of Stockholm, and the southeast rural area of Sweden. Each location presents unique obstacles to continuity of care, influenced by factors such as population density and geographical disparities. By examining the experiences of patients with complex care needs, their family carers, and healthcare personnel, this study aims to describe the conditions for and identify potential solutions associated with the delivery of continuity in care in different geographical regions of Sweden, with differing population densities.

**Method:**

Secondary analysis was conducted using qualitative content analysis on interview data from two studies, consisting of 53 transcripts from individual, pair, and focus group interviews held between August 2018 and November 2019. The potential solutions identified from participants’ experiences were categorized into region-specific and common themes. Three personas—Vera, Bo, and Inga—were developed, each representing a scenario based on the region-specific analyses.

**Results:**

Despite regional differences, universal solutions to common challenges were identified focusing on relational, management, and informational aspects. Common key obstacles to continuity of care included resource shortages, insufficient information transfer, and privacy regulations. Possible solutions for overcoming these challenges include prioritizing relational continuity, streamlining processes, and advocating for a unified communication system. By collaborating, building trust, understanding patient preferences, and ensuring clear communication, healthcare personnel can effectively promote continuity of care.

**Conclusions:**

Building a stable workforce while prioritizing relational continuity, along with patients’ preferences and needs, is essential for ensuring continuity of care from multiple providers. Digital solutions can enhance collaboration across distances, while coordinating responsibilities within smaller geographical areas can strengthen partnerships among healthcare organizations. Direct dialogue, along with ensuring that everyone has access to relevant information through a unified communication system, is vital for management continuity. By integrating these universal and transferable solutions to the obstacles associated with continuity of care, we can create a cohesive care experience for patients, regardless of geographical and demographic conditions.

**Supplementary Information:**

The online version contains supplementary material available at 10.1186/s12913-025-12649-1.

## Background

Continuity of care is widely recognized as a crucial component of high-quality care, contributing to increased safety [50], more efficient and person-centered care, and greater patient satisfaction [46]. It enhances collaboration among a patient's care contacts [13] and reduces the risk of polypharmacy and gaps in care [14, 16]. It also leads to better health outcomes [41], ensures timely and appropriate care [31], and lowers the rate of hospital visits and the mortality risk [31, 37]. Numerous studies have demonstrated that continuity of care provides patients with a sense of security, consistency, availability, and confidence, resulting in higher levels of satisfaction [16, 21, 24, 40, 49]. Patients experience continuity when management continuity is ensured through well-functioning coordination of care during transitions [24, 36] and information transfer (informational continuity) is adapted to the patients’ needs and includes both past, present, and future. Relational continuity builds a holistic understanding through mutual interaction and trust [36]. Knowledge continuity occurs when information is integrated with knowledge, and a mutual understanding is developed regarding ongoing and planned care and treatment [34]. The concept of continuity of care encompasses the patient's multidimensional perspective, which can evolve depending on the patient's resources, needs and conditions [36]. Continuity of care forms the foundation for patient satisfaction with care as it serves to enhance person-centeredness [17]. It also aligns with the perspectives of healthcare managers’ goals of effectiveness, and efficiency [22, 23]. This improvement in patient experience and reduction in costs are part of the triple aims, contributing to the achievement of the primary goal of population health [4]. Additionally, relational continuity has the potential to address the 'quadruple aim' by enhancing the well-being of healthcare personnel, as it has been shown to improve their work satisfaction.

A challenge for people living with chronic illnesses is that this often necessitates contact with multiple healthcare workers, at least one for each illness [38]. Patients and their family carers need to navigate the system and coordinate care and treatment from the different healthcare providers. One critical challenge is inadequate information sharing and coordination between healthcare providers, which often leads to incomplete patient treatment plans and creates risks of delays, errors, omissions, or incomplete care [18, 33]. In addition, high-quality care relies heavily on the frontline staff working directly with patients. Healthcare personnel handle daily safety risks and ethical dilemmas, making their access to adequate information, continuity, competence, and appropriate resources crucial to meeting demands [12].

Potential solutions to improve continuity of care include implementing various integrated care models. These models aim to reduce fragmentation and enhance both continuity and safety in healthcare. The High-Quality and Local Healthcare reform [47] was implemented in Sweden to reduce fragmentation and promote cooperation between regions and municipalities. The focus areas are creating a strategic entry point and improving access to health services, developing long-term personal relationships, ensuring a range of services and collaboration between these services, and promoting person-centered care. To create continuity of care, the individual care plan has been introduced. This describes the patient's past, present, and future by following the patient's care trajectory and giving everyone involved the information they need [39]. Care planned together with patients and family carers can be knowledge-based and organized based on each patient's needs and conditions [47].

Although efforts have been made to improve continuity in care, challenges persist, including limited availability and long waiting times for certain services, gaps in coordination between care providers, and inconsistencies in aftercare follow-up. In Sweden, as in many other countries, access to care varies across regions, leading to different experiences in different geographical locations. Since care must be flexible and personalized to individual needs and cultural background, which greatly impact the level of support required, it is essential to identify potential solutions that are both universal and transferable. Thus, this study aims to describe the conditions for and identify potential solutions associated with the delivery of continuity in care in different geographical regions of Sweden, with differing population densities.

## Method

### Design

The study has an inductive, descriptive, and qualitative secondary research design. Secondary analysis of existing datasets is increasingly recognized as an effective method to maximize knowledge and provide valuable insights to policymakers and health services [25, 52].

### Settings

The secondary analysis was based on data from two studies conducted in three different geographical regions in Sweden, which has 10.5 million inhabitants living in 21 regions [36]. The northern regions, Norrbotten and Västerbotten, occupy approximately 40% of Sweden’s land area and have a total population of about 0.5 million, 5% of the Swedish population. Most inhabitants live on the east coast, whereas the inland is very sparsely populated, with approximately 1 inhabitant/km^2^. One group of informants in this study were from the inland. The capital city Stockholm, located in the southern third of Sweden, on the east coast, is the most highly populated region with just over 20% of the country’s population, about 2.4 million inhabitants, and a population density of 373 inhabitants/km^2^. This was the second area included in this study. Further southeast of Stockholm is the Kalmar region. This was the region with the smallest population in the study, just over 247,000 inhabitants and a population density of 22 inhabitants/km^2^. This region encompasses Sweden’s second-largest island, Öland.

Healthcare provision faces different conditions and challenges in these dissimilar regions. In Norrbotten and Västerbotten, one major challenge is the long distances between inhabitants and healthcare centers and social care services. Inhabitants may have to travel up to 300 km to the nearest hospital with specialist functions and there is sometimes a lack of competences. Healthcare personnel working in sparsely populated regions face other challenges regarding continuity of care than those working in large cities. The problem is not that there are few people in a large area, but rather how to create a well-functioning and robust labor market and run public and private services cost-effectively [32]. In Stockholm, the challenges are of a different kind– with many specialist hospitals and healthcare services, both public and private providers, there are challenges to informational continuity when several different healthcare providers are involved. Several municipalities in both Region Västerbotten, Norrbotten, and Kalmar have more than 30% of the population aged 65 years or older.

### Participants

Participants in the studies were patients with complex care needs, family carers, and healthcare personnel (Table 1) recruited from hospitals, home healthcare, or healthcare centers, to capture a broad perception of continuity of care. Inclusion criteria for patients were having complex care needs, i.e., suffering from one or more chronic illnesses, and having two or more health or social care contacts [51]. Family carers were defined as relatives providing some degree of care, assistance, or support– emotional, logistic or financial – and were selected by the patients. Healthcare personnel had to have experience and knowledge of health and/or social services. The purpose was to include a wide variety of care providers and professions, with as different roles and perceptions as possible. For further description of the studies, see Table 1.

**Table 1:** An overview of participant characteristics

**Patients with complex care needs**	**N**
***Sex*** (n)	
Male	7
Female	9
***Age*** (years)	60–95
***Diseases***	Stroke, chronic obstructive pulmonary disease, diabetes, kidney failure, heart failure, cancer, atrial fibrillation, high blood pressure, polymyalgia rheumatica, lymphoma, goiter, Sjogren’s syndrome

**Family carers**	**N**
***Sex*** (n)	
Female	10
Male	2
***Relationship to the patient***	
Wife	4
Daughter	3
Brother	1
Husband	1
Sister	1
Daughter-in-law	1
Ex-wife	1

**Healthcare personnel**	**N**
***Sex*** (n)	
Male	2
Female	32
***Care provider***	
Municipal care^a^	18
Primary care^b^	14
Specialist care^c^	3
***Profession***	
Registered nurse	16
Assistant nurse	9
Physiotherapist	4
Physician	3
Social worker	2
Occupational therapist	1

^a^home care, home health care, social services

^b^Public primary care providers

^c^Advanced home health care department and hospital

### Data collection

Interviews used in this study were collected for two earlier studies [35, 36]. The first aimed to describe aspects of continuity of care that patients and family carers considered essential. The second aimed to investigate healthcare personnels’ perceptions of the prerequisites for continuity of care within and between organizations and how continuity of care can be realized for patients with complex care needs. Individual, pair, and focus group interview were conducted in August 2018–November 2019. The semi-structured interview guides used were developed for these earlier studies and have been previously published elsewhere [35, 36]. The interviews were performed at the participant’s home or workplace, at a healthcare center or hospital, or by phone, and lasted approximately 17–60 minutes. Each interview was audio-recorded and transcribed, and the transcribed interview text was de-identified. In total, 53 transcripts were included.

### Analysis

**Fig. 1:**
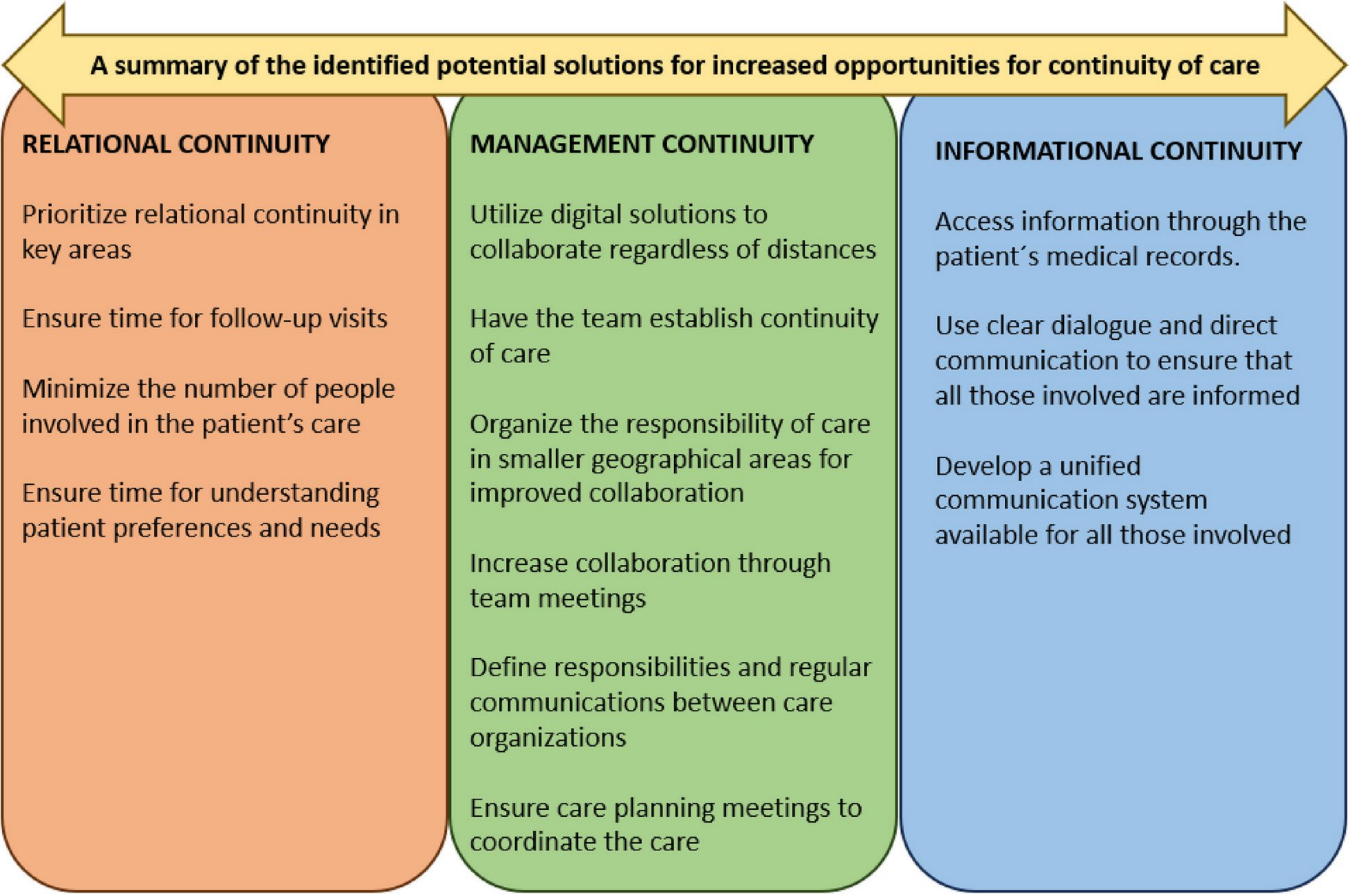
An overview of similar and different potential solutions for continuity of care from the perspectives of Vera, Bo, Inga, their family carers, and their healthcare personnel related to the dimensions of continuity

A secondary analysis of existing interview data [7] was performed through qualitative content analysis [19, 20]. The analysis started by 1) reading the uncoded transcribed interview texts several times to get familiar with the data. Thereafter, 2) meaning units such as sentences that corresponded to the aim were marked in the text. The meaning units were then placed into an Excel sheet and 3) condensed, with the goal to reduce the number of words but retaining the main content. 4) The meaning units were coded, meaning that a few words were used to describe the content. These descriptions were kept close to the interview texts, to show variances in the data. 5) Similar codes were then sorted into categories [20]. The category names were described with a low degree of interpretation [19]. 6) The categories were analyzed based on which of the included regions in Sweden the included codes came from. The categories that contained codes from only one region were sorted into region-specific groups (a, b, c) highlighting the differing conditions and solutions in the three areas (for overview see Table 2). The remaining categories, which shared similarities in conditions across areas, were sorted into a common group. This group was then divided into subgroups to address the relational, management, and informational dimensions of continuity (overview categories Table 3). Potential solutions described through the participants’ experiences were identified in both the region-specific categories (differences) and common categories (similarities). These solutions were then sorted together and abstracted, see Fig. 1. Three personas, Vera, Bo, and Inga, were created, each with a specific scenario. Each persona was based on the analysis of the region-specific categories, and assigned to its specific region (a, b, c). The goal of using personas and scenarios was to facilitate a detailed, concrete, comprehensive description of the results and to visualize the complexity of the aim from different perspectives. The use of personas deepens the understanding of, for example, conditions and solutions for continuity of care [1, 6, 28]. An AI-generated picture was created for each persona using DALL-E2. The results were then described at a manifest level from the perspectives of the personas. Quotes are presented in the supplementary material, to strengthen and contextualize the results.

**Table 2:** An overview of the region-specific differences in conditions and potential solutions described in each scenario

	**Sparsely populated area from the experiences of VERA, her daughter, and her healthcare contacts**	**Urban area from the experiences of BO, his daughter, and his healthcare contacts**	**Rural area from the experiences of INGA, her husband, and her healthcare contacts**
**Conditions for continuity of care**	Missing the personal care and familiarity provided by a dedicated general practitioner (GP)* Dependence on the family carer and community for continuity of care* Dependence on stability among healthcare workers to compensate for the lack of GPs** Limited collaboration with healthcare providers across regional borders**	Challenging to navigate multiple care organizations and appointments* Lacking someone with an overarching responsibility** Lacking opportunities to maintain relational continuity**	Confident in knowing who to contact*** Challenging and time-consuming to access and review care plans**
**Potential solutions associated with continuity of care**	Enhancing continuity in care by: Utilizing digital solutions to bridge gaps in care**	Enhancing continuity in care by: The team establishing continuity of care** Limiting care responsibility to smaller geographical areas for increased collaboration**	Enhancing continuity in care by: Increasing collaboration through team meetings**

*Vera’s, Bo’s, Inga’s, or their family carers’ experiences

**The healthcare workers’ experiences

***Everyone’s experiences

**Table 3:** An overview of categories on conditions and potential solutions that are similar across regions, divided into subgroups to address relational, management, and informational dimensions of continuity

	Categories consistent between two or all regions based on the experiences of Vera’s, Bo’s, Inga’s, their family carers’, and the healthcare personnel related to dimensions of continuity		
	Relational continuity	Management continuity	Informational continuity
**Conditions for continuity of care**	Beneficial with relational continuity in care** Beneficial to meet the same healthcare workers* Challenging to meet different healthcare workers* Beneficial to meet different healthcare workers*** Lack of personnel hinders relational and management continuity**	Challenging to collaborate between care organizations*** Time-consuming to collaborate and coordinate care***	Having sufficient knowledge about health and care plan* Challenging to obtain information*
**Potential solutions associated with continuity of care**	Enhancing relational continuity by: Prioritizing relational continuity in key areas** Ensuring time for follow-up visits** Minimizing the number of people involved** Taking the time to understand each patient's preferences and needs**	Enhancing management continuity by*:* Defining responsibilities and regular communications** Care planning meetings***	Enhancing informational continuity by: Relying on the patient’s medical records** Clear dialogue and direct communication** A unified communication system**

*Vera’s, Bo’s, Inga’s, and their family carers’ experiences

**The healthcare workers’ experiences

***Everyone’s experiences

## Results

The findings are presented through three personas, Vera, Bo, and Inga, with region-specific scenarios, reflecting a sparsely populated region, an urban region, and a rural area. First, findings describe *differing* conditions and solutions related to continuity of care depending on living location (Table 2). Secondly, in contrast, findings describe similarities in conditions and solutions that are independent of the living situation (Table 3). Both parts include the perspectives of patients, family carers and healthcare providers. The commonalities and differences in conditions and potential solutions are contrasted against three major dimensions of continuity of care (Table 3, Fig. 1) to address the study aim.

### Personas with scenarios based on region-specific characteristics

#### Vera’s scenario (sparsely populated area)


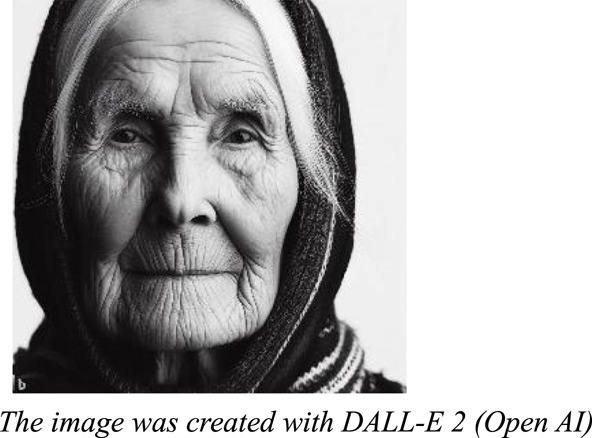


*Vera is a 90-year-old woman who lives alone in a house in a sparsely populated region. She has several diseases and medications. Vera experiences difficulties moving around and does not have a driver's license. To manage her daily activities, she receives home healthcare and social services three times a day. She receives additional assistance from her daughter, who lives in a house nearby, and her neighbors, who help out with practical tasks around her house.*

The unique conditions in Vera’s scenario are that she and her daughter have historically valued the *personal care and familiarity provided by a dedicated general practitioner (GP)*, but due to the shortage of GPs, they now face challenges in accessing consistent care. Vera *depends on her daughter, the community, neighbors, and healthcare personnel* other than a GP for support and continuity in her healthcare journey. Vera´s healthcare providers recognize the need to compensate for the lack of a GP by promoting *stability among other healthcare personnel*, such as registered nurses (RNs) and assistant nurses. Although they experience good collaboration within their own region, they feel that *collaborations with healthcare providers across regional borders are limited*. A unique solution in Vera´s scenario is that healthcare providers *sometimes utilize digital solutions to bridge gaps in care*, especially when accessing specialized services from distant hospitals in other regions. Despite obstacles, they strive to maintain relational and informational continuity in Vera's healthcare experience and enhance communication about it.

#### Bo’s scenario (urban region)


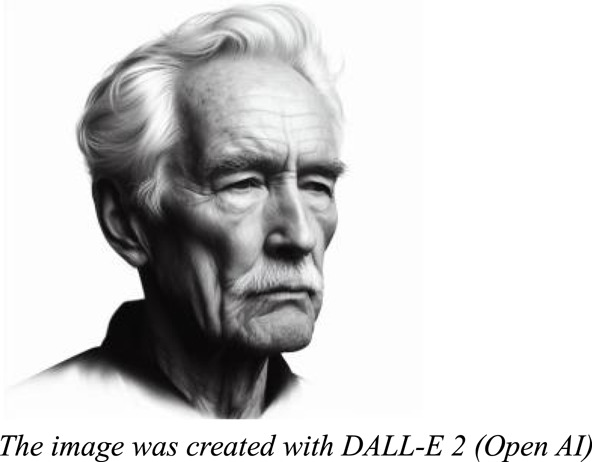


*Bo is an 80-year-old man who lives alone in an apartment in an urban city. He has several diseases and medications. Bo experiences difficulties with mobility and his health conditions now prevent him from driving, although he has had a driver’s license. To manage his daily activities, he receives home healthcare and social services twice a day. His daughter lives in the same city and assists him with healthcare contacts, among other things.*

The unique conditions in Bo´s scenario is that he and his daughter find it *challenging to navigate multiple care organizations and appointments*, often feeling confused about the purpose of various assessments and appointments. They feel that the constant travel and lack of coordination between providers is a waste of time and energy. Furthermore, Bo's healthcare contacts also struggle with the *lack of a person with overarching responsibility* for his care, as this results in gaps in communication and treatment. They believe that overlapping responsibilities and use of digital systems could help provide a more comprehensive overview of Bo's care. Bo's GP experiences a *lack of opportunities to maintain relational continuity* in a large healthcare system. The unique solutions in Bo´s scenario is to work in *teams which can help to enhance relational continuity* by reducing the number of healthcare workers involved in Bo's care. The team's goal is to develop a holistic understanding of Bo's needs and prioritize consistent care over familiarity with providers. Moreover, they believe that *limiting care responsibility to smaller geographical areas* would also enhance collaboration with other providers and continuity.

#### Inga’s scenario (rural region)


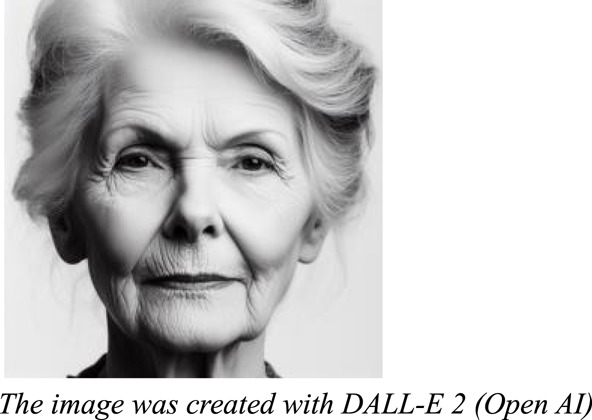


*Inga is an 85-year-old woman who lives together with her husband in a house in a rural region. Their three children live in other parts of the country, far from Inga. Inga has several diseases and medications. She has difficulties moving around, and her husband supports her daily activities. After Inga’s latest hospital visit, she started receiving home healthcare and social services three times a day.*

The unique conditions in Inga’s scenario is that she and her husband are satisfied with the level of home healthcare they receive and feel *confident that they know who to contact* for assistance. The healthcare personnel involved in Inga's care appreciate having one designated RN responsible for each nursing home and home healthcare group, which streamlines communication and coordination. Additionally, a dedicated contact person was assigned to assist Inga after her hospital discharge, facilitating a smoother transition and continuity of care for a month. However, the healthcare personnel find it *challenging and time-consuming to access and review Inga's care plans*, as she has been treated in different regions with different medical record systems. There is written patient information available in Inga's home, but they struggle to find the time to review it thoroughly.

The unique solution in Inga’s scenario is that *team meetings are seen as beneficial for enhancing collaboration* among the healthcare personnel involved in Inga's care. These meetings provide a platform for sharing knowledge, clarifying roles and responsibilities, and improving communication and coordination. The key to successful team meetings lies in having a designated coordinator and maintaining a structured format. Overall, Inga's healthcare personnel value the benefits of teamwork and effective communication in providing quality care for their patient.

### Similarities in Vera’s, Bo’s, and Inga’s scenarios related to the dimensions of continuity of care

*Vera, Bo, Inga, their family carers, and healthcare personnel share common experiences across different regions regarding addressing and improving relational, managemental, and informational continuity of care. (for overview categories see* Table 3 and Fig. 1).

#### Relational continuity

The similarities in all scenarios were that all involved recognize the *value of relational continuity in care* for better outcomes and a more efficient healthcare experience. Vera, Bo, Inga, and their family carers find it *beneficial to consistently meet the same healthcare workers,* as it creates a sense of security, respect, and familiarity. This continuity of care strengthens their confidence in the treatment they receive. *Meeting different healthcare workers is challenging* for them, as it can lead to confusion, lack of trust, and a disjointed approach to care. Although there are instances where *meeting new healthcare workers may be beneficial*, as they offer different perspectives, the overall preference is for consistency, providing a holistic and personalized approach.

Vera’s, Bo’s, and Inga’s healthcare workers, like many others, are facing a shortage of resources in their field. The *lack of GPs and RNs is posing challenges to maintaining patient relationships and coordinating care* across different healthcare settings. To overcome these obstacles, they are focusing on *prioritizing relational continuity in key areas* such as intimate care and for patients with more complex medical needs. They are also streamlining their processes by *minimizing the number of people involved* in patient care, to improve the overall quality of patient-provider relationships. Moreover, they strive to take responsibility for *enhancing relational continuity by booking follow-up visits* and participating in care planning to stay informed and meet their patients' needs. Vera, Bo, and their family carers find follow-up visits to be valuable, as they feel safe and well-informed about their care plans. The healthcare workers recognize that follow-up meetings are essential for establishing continuity of care. Vera’s, Inga’s, and Bo’s healthcare workers want to prioritize *taking the time to understand each patient's preferences and needs*, emphasizing the importance of building trust and ensuring that their patients feel secure. They value open communication, to gather knowledge of their patients' wishes and needs, enabling them to provide personalized care.

#### Management continuity

Vera, Bo, Inga, and their family carers are facing *challenges in collaboration between healthcare organizations* due to poor knowledge transfer and lack of interest from healthcare personnel. Healthcare workers also struggle with limited collaboration possibilities, lack of routines, inadequate information and communication technology, and limited knowledge of each other's work. A more seamless and efficient collaboration between healthcare organizations is needed to improve the care provided to Vera, Bo, Inga, and others.

Bo's and Inga's family carers and healthcare providers find that *collaboration and coordination can be time-consuming*. Bo's and Inga's family carers often have to make multiple calls to schedule appointments with physicians, whereas healthcare personnel struggle to connect with each other. Hospital healthcare personnel, in particular, find it challenging to participate in care planning meetings due to competing priorities. However, Vera's and Inga's healthcare providers benefit from organizational strategies that improve continuity of care, such as *clearly defined responsibilities and regular communication with other healthcare organizations* in the region. Collaboration is seen as essential in achieving optimal care for Vera, Bo, and Inga– and healthcare personnel are willing to invest time in working together. *Care planning meetings before discharge are found to be particularly effective in enhancing collaboration* and ensuring that all parties are on the same page regarding care plans. Overall, healthcare personnel and family carers alike appreciate the benefits of coordinated care and clear communication in providing quality care to Vera, Bo, and Inga.

#### Informational continuity

Vera, Bo, Inga, and their family carers navigate a complex healthcare system where they *have important knowledge* about their health and care plans. They are proactive in seeking the care they need, but at times struggle to have the strength to communicate effectively with healthcare personnel, understand the information provided, and access comprehensive care details. It is *challenging for Vera, Bo, Inga, and their family carers to obtain information* about care plans, for example, and there is a lack of written summaries and pharmacy lists. Similarly, it is challenging for some of their healthcare workers to get information from other healthcare providers. Vera’s, Bo’s, and Inga’s healthcare workers value the importance of informational continuity in patient care. They often *rely on patient medical records* to communicate with each other and stay updated on their patients' care. However, they face challenges in accessing medical records due to different systems being used by different providers. They advocate for a common system to facilitate seamless information exchange. The families’ primary source of information remains their physician or community health nurse, and one of the healthcare providers’ sources of information is always the patient and any family carer.

To bridge the gap, the healthcare personnel prioritize *clear dialogue and direct communication* to ensure everyone involved in the care plan is informed and on the same page. They go the extra mile by reaching out through calls or letters to share vital information with other healthcare organizations.

Despite the limitations, they see the *benefits of a unified communication system* where all the personnel involved can access and share information easily, especially when patients are hospitalized or being discharged. They believe this would improve collaboration and enhance the overall quality of care for Vera, Bo, and Inga.

### Methodological considerations

Ethical considerations have consistently been reflected upon throughout the study, and no sensitive personal data were handled during the secondary analysis. The study’s aim aligned with the phenomenon regarding which participants had given written and verbal consent prior to the interviews. The benefit of secondary analysis, which maximizes participants’ contributions, is judged to outweigh potential ethical risks [7].

The study has both weaknesses and strengths that need to be considered. A weakness of the study is that it was a secondary analysis– the interviewers did not have the aim in mind during the interviews and therefore were unable to ask follow-up questions related thereto. However, the aim of the primary studies was within the same phenomenon as the present studies, and the interview guide covered a broad range of questions that were largely connected to the aim of the secondary analysis. Moreover, there were no significant changes in setting or routines from the time of the interviews to the present study.

A strength of the study is the inclusion of what is, for a qualitative study, a relatively large number of participants, with different roles in different parts of the healthcare organization. Their experiences are judged to provide knowledge of the phenomenon from various perspectives, thereby offering a more comprehensive understanding of the aim. A further strength is that the interview data were uncoded, to minimize the risk of being influenced by previous analyses. Moreover, the analysis was performed by the last author, who was not involved in the primary studies, thus minimizing the risk of bias [25].

The similarities found, independent of location, are intriguing as they suggest that similar approaches to solutions may be applied, potentially allowing for the transferability of the results across different contexts. In contrast, the region-specific categories in the data may reflect the unique participants to varying extents, rather than being specific to a region. However, the data provide a snapshot, with no claim made that they present constant differences. By including different regions and describing aspects that are specific to each, it is possible to gain and transfer knowledge between them and similar regions. The possibility of transferring knowledge from this study to other settings is best judged by the reader [42].

## Discussion

The results show that there are both unique and shared conditions for continuity of care across various contexts, particularly in terms of resources, information transfer, and collaboration. However, the results show that the potential solutions described are both tailored to the specific context while also exhibiting commonalities across different areas. As continuity in care is experienced as multidimensional, with essential aspects such as relational continuity, information sharing, and management working in synergy [36], the results will be discussed in the context of this dynamic interaction.

Vera, Bo, Inga, and their family carers preferred meeting the same healthcare personnel, regardless of regional differences. Healthcare personnel also valued such continuity, as understanding each patient's preferences and needs is time-consuming. Relational continuity is essential for healthcare personnel, patients, and family carers to integrate information, create knowledge, and develop a shared understanding [11, 34]. This study highlights that enhancing relational continuity often requires taking personal responsibility. Healthcare personnel’ handling of relational continuity is similar despite the challenges differing between regions. However, the biggest challenge in all regions was having the possibility to meet the same physician, especially in case of repeated appointments. In this concern, region-specific differences occurred, mainly by addressing management solutions. In sparsely populated areas, RNs and assistant nurses tried to maintain relational continuity to offset the lack of physicians. Bo experienced healthcare in the urban city as fragmented, being shuffled between different care organizations that addressed one condition at a time. Healthcare personnel in the urban city argued that there should be overlapping responsibilities among care organizations and digital systems, to ensure a comprehensive view of patient care as a potential solution. They also suggested that smaller teams could enhance continuity by reducing the number of caregivers involved in Bo's care. With this approach, relational continuity could develop within a team of carers rather than relying on a single dedicated person. This could be a common solution for all regions, making relational continuity more achievable. Similarly, healthcare personnel caring for Inga, in the rural region, found that working in teams was essential. They noted that regular meetings enhanced collaboration and helped participants understand who was involved in Inga's care and their respective responsibilities. Interprofessional collaboration (IPC) allows healthcare workers from different disciplines to work together, share knowledge, and coordinate care without strict hierarchical structures [10]. This approach enhances access and coordination of services, particularly for chronic conditions, and is suggested to reduce complications, readmissions, and clinical errors [43, 44]. However, the development of IPC team-based competencies in municipal and home healthcare settings is still rather unexplored [29].

A common experience across regions in the current study was that informational continuity was enhanced through dialogues and by having access to a common communication system during discharge from hospitals. For most healthcare personnel, IPC involves daily, informal, sometimes unplanned, clinical and non-clinical interactions. Maintaining effective collaboration across healthcare organisations is challenging independent of location. A potential solution for maintaining informational continuity of care is IPC. There is evidence that IPC can positively influence patient, healthcare personnel, and organizational factors in hospital settings, but challenges to IPC have also been revealed [8, 43]. Inadequate information, especially when some disciplines are underrepresented, can skew treatment goals and team collaboration can suffer due to differing perspectives on care, hierarchical structures, and power imbalances [8, 26]. Deficiencies in information management not only result in a lack of informational continuity, but also pose risks to patient safety [27].

Healthcare personnel in the current study faced obstacles in care management due to the lack of common communication systems, limited collaboration, and restricted access to patients' medical records across organizations. These issues were mitigated by the solutions of building familiarity between organizations, knowing who to contact, and holding care planning meetings, although such solutions were time-consuming. However, these care management solutions might be easier in sparsely populated and rural regions. Incompatibility of information technology systems has been shown to hinder information continuity [3]. Additionally*, insufficient access to information* leads to difficulties in obtaining an overview of a patient's care and creates a power imbalance between the healthcare workers, the patient, and the family carers. It limits the patient’s participation and decision-making in their care, potentially resulting in unequal treatment [11]. Developing access and transfer of information both verbally and in writing is a potential solution for increasing continuity of care. This is also supported by Khatri et al. [30], who argue that to increase informational continuity, effective communication channels and a formal decision-making structure supported by various functions are needed. However, there are barriers in the healthcare system– such as data ownership and confidentiality– that prevent information sharing and related information transfer.

Similarities among Vera, Bo and Ingas experiences were that they experienced a lack of information, especially written information. They addressed this by personally ensuring the transfer of necessary details. Patients and family carers become an important part in bridging the gaps and maintaining continuity of care by carrying patient information and creating coordination. However, patients may not remember information about their care, and family carers may not themselves have gained sufficient information. Not having sufficient information, but being expected to provide care, increases the burden on family carers [5]. Studies show that family carers’ burdens affect both their health, their social life and their work life [15]– financially, physically, and emotionally [48]. The need to address barriers to informational continuity for all involved is largely connected to management. Ensuring a unified communication system is available for patients, family carers, and all significant people involved in care to receive written information should be a primary concern for healthcare management.

A proactive bridging of systemic deficiencies was shown by healthcare personnel across the various healthcare settings and regions. The successful delivery of seamless care is suggested to rely on three key factors: understanding the patient, knowledge sharing across the interprofessional team, and proactively bridging gaps within the system [2]. We highlight a fourth key factor: dynamic stability within organizations [35]. Such stability enables information transfer between different levels of care and healthcare providers (both horizontally and vertically in the system) and is supported by leadership that fosters trust and involvement, ultimately enhancing the well-being of healthcare personnel, patients, and family carers [45].

## Conclusion and implications

In conclusion, collaboration between healthcare organizations, and effective communication between healthcare personnel, the patient and family carers is essential in providing high-quality, personalized care for patients like Vera, Bo, and Inga. Although regional differences exist, universal solutions to common challenges were identified. Challenges to continuity of care included resource shortages, lack of information transfer, and privacy regulations. Healthcare personnel are committed to overcoming these obstacles by prioritizing relational continuity, streamlining processes, and advocating for a unified communication system. By working together and focusing on building trust, understanding patient preferences, and ensuring clear communication, healthcare workers and family carers can ultimately enhance the overall healthcare experience and improve outcomes for patients. Continuity of care, collaboration, and communication are paramount in providing quality care for patients like Vera, Bo, and Inga. By integrating these identified universal and transferable solutions to the obstacles associated with continuity of care, we can create a cohesive care experience for patients, regardless of geographical and demographic conditions.

## Supplementary Information

Supplementary Material 1. Relational continuity: Quotes showing similarities in Vera’s, Inga’s and BO’s scenarios. 

## Data Availability

The datasets analysed during the current study are not publicly available as per the Regional Ethical Review Board’s guidelines to protect the privacy of the participants but are available from the corresponding author on a reasonable request.
