# Supplementary material for: Conditions for and potential solutions associated with continuity of care for patients with complex care needs across Swedish regions with differing population densities
Source: BMC Health Serv Res. 2025 Apr 28;25:614. doi: 10.1186/s12913-025-12649-1 (PMC12036265; doi:10.1186/s12913-025-12649-1)
Supplement: Supplementary file 1 — Supplementary Material 1. Relational continuity: Quotes showing similarities in Vera’s, Inga’s and BO’s scenarios. [file 12913_2025_12649_MOESM1_ESM.docx]

Appendix 1. Quotes from the participants in each category, divided into scenarios

QUOTES FROM VERA’S SCENARIO

**Missing the personal care and familiarity provided by a dedicated general practitioner (GP)**

*‘And then you got to meet the same person every time and she knew exactly what the status was last time and so on. Well, but now she’s retired, so I don’t know…no, I don’t really know who I’ll be seeing…’* VLLP4

*‘So that’s what I think is lacking with the temporary doctors. Back then, we had doctors working here full time, and they knew every person living here too and could joke around with you. It was a completely different thing. But now you might get someone from Iceland or Norway or Iran and they can be as nice as you please…but there’s no continuity.”* VLLN3

*‘And some weeks this summer, we haven’t had any temporary doctors, no doctors on site in X for…several weeks. I mean, the first time it was for a week and the second time it was two weeks that we didn’t have a doctor in X. So…you better not get sick.’* VLLN4

**Dependence on the family carer and community for continuity of care**

*‘…I have my network here, I feel. Because they know…if some neighbour sees that there’s no smoke from the chimney or when the light comes on in the morning, they’ll call to say “Did you sleep in today?” or something like that. You don’t get that in a bigger city.’* VLLHP2

**Dependence on stability among healthcare workers to compensate for the lack of GPs to ensure continuity of care**

*‘…so now we have fixed nursing staff, and the difference is noticeable, because now we’re the ones who know the patients and the doctors will say “since you know them, can you help me?”.’* VLLHP4

*‘But for us…our vocational nurses are the same, so they know our patients really well and can say “oh, don’t you remember, it was, it’s this…it’s that…” …and can give information to someone who maybe hasn’t met the patient.’* VLLHP6

**Limited collaboration with healthcare providers across organisations and the regional border**

*‘…here in town, locally, I mean, we have a very close collaboration with the municipality and home care and so on. Then, if you think about…healthcare providers within the region…then it’s more varied depending on the specific unit, I have to say. But I often feel that they pass the buck… “that isn’t my responsibility, that’s primary care” and so on. So, I feel that the collaboration is more lacking… Because in our case, you have to think about the distances too, in my opinion.’* VLLHP3

**Utilizing digital solutions to bridge gaps in care**

*‘I called the on call at plastic surgery and that worked…I was in touch with them within ten minutes. I said “I have a huge issue with a burn, could you look in the media archive, I don’t know what to…” Then they looked and said “the patient needs to come down, that doesn’t look good, send the patient down here.” Boom, the patient went down and had surgery two days later. It’s healed now, so sometimes good things happen.’* VLLHP4

QUOTES FROM BO’S SCENARIO

**Challenging to navigate multiple care organisations and appointments**

*‘And then I’ll meet a doctor, and then they’ll send me to someone else, that’s been the case the entire time…and I still don’t know what it is. That makes you lose interest in yourself.’* SLLP3

*‘I think they do quite a lot of tests that are unnecessary…so it’s a waste of both resources and time, and his time and energy, because he can’t always be going here and there.’* SLLN1

**Lacking someone having an overarching responsibility**

*‘Other healthcare providers do different tests three days in a row because there are different dates on them…and then we’ll suggest that we do them all at once and they’ll be surprised. No one looks at that when they make bookings in the system, they just book their own thing. But I think there should be something when multiple healthcare providers are involved, so they have to coordinate…that you can’t order a test before you check if there are other orders already.’* SLLHP3

*‘…and then that you could work more like a network, that you have to overlap each other in a different way, I think, and feel some responsibility for that, instead of each person doing their thing.’* SLLHP5

**Lacking opportunities to maintain relational continuity**

*‘I don’t think you can have that, like, classic continuity with the doctor knowing every single patient, I don’t think that’s possible…when I started working thirty years ago, I could manage a lot on my own, but now it feels like you have to check more and more things with some healthcare worker or other, and then you don’t have the chance… It’s just not possible, the way primary care is now, to meet the same patient, so you have to find other ways to get good continuity.’* SLLHP6

*‘Yeah, because if I access the website to book an appointment with XX and he’s not even on the list, well, then I have to choose some other doctor, because I need help.’* SLLP1

*‘Sometimes the patient needs home healthcare when we’re finished treating them. Then we might find that not all healthcare centres offer home healthcare, so you have to choose another centre. And that’s not always welcomed by the patient, because maybe the patient wants to keep going to their doctor at the healthcare centre and see their district nurse, but doesn’t have the energy to get there. They need home healthcare and then some other unit has to step in. So, that’s hard.’* SLLHP4

**The team establishing continuity of care**

*‘I think the best thing is really to get the patient to feel secure that…no matter which nurses are there, they’ll be providing the same care…not randomly based on what I think is best…it should be the same process each time.’* SLLHP2

*‘…it doesn’t have to be the same person, and just that there’s a good team around that…and that they feel secure in that…there’s someone there for them or someone familiar with the patient’s situation …’* SLLHP6

*‘We talk a lot about creating continuity in some other way than centred around an individual. It has to be, so to speak…organisational continuity.’* SLLHP3 sskchef ASIH

**Limiting care responsibility to smaller geographical areas for increased collaboration**

*‘If you had your areas. If the healthcare centre’s staff were also divided into the same areas, and the home care and the social workers, that would be great. Then we could solve a lot of issues quickly, without having to employ more people to work with organising things.’* SLLHP7

*‘Yeah, but it’s easier to collaborate if there’s a personal touch, that you know something about the people, so maybe it’s easier to get the conversation to work. It seems that way.’* SLLHP3

QUOTES FROM INGA’S SCENARIO

**Confident in knowing who to contact**

*‘I have a good contact with the doctors…I’ve been in touch with [town name], I know the rheumatologist there, so I felt it was easy to call them if anything came up.’* KLLP7

*‘But you know that’s a safe space and if anything comes up, you can call home care…and that’s…there’s a sense of security in that.’* KLLN5

*‘We have nurses who have responsibility for the patients in each housing unit, each department and each home care group. That’s their continuity, that they know we have X here and then XX is responsible for them, so that’s who we should contact. And I think that works well.’* KLLHP7

*‘And then we have this thing with Safe return home, and that’s like a guide, who works with the transition and the home care, during the period from when the person returns home and for about a month. They’ll be waiting on the steps when the person returns from the hospital. And that’s the contact who makes things feel safe for the person coming home from hospital. They should know that this is a person they can turn to, whatever type of help they need.’* KLLHP4

**Challenging and time-consuming to access and review care plans**

*‘Our medical record system is kind of…I don’t think you get a good overview. Everyone writes notes in the record. Doctors, nurses, the physical therapist, everyone, and it’s in chronological order, so if the patient has been in hospital for a week, you might have 20–30 notes, easy…at the end there’s a discharge summary that might not be optimal, so it’s hard to get an overview of what has happened.’* KLLHP6

*‘If I go to the home of a patient who I’ve never visited before, and it’s very detailed and clear what I’m supposed to do in order for them to feel that they get the same help and care regardless of who is visiting them, then there’s a plan behind that. But many people feel that they don’t have time to read a performance plan in the morning. If you’re visiting five people that morning, then you don’t have time to study all those plans…and if you’re coming in as a temp and have to stand in the hallway and try to read it. How’s that supposed to work?’* KLLHP5

**Increasing collaboration through team meetings**

*‘I really value the team meetings. When we got started with the teams, it was a real eye-opener, like “oh, so you do this, that’s what we’re supposed to use you for”…and we would collaborate with the nurse in a sensible way. A forum like that is much better, that we are in contact with each other more. I’d like to have more of that kind of forum with the various units.’* KLLHP3

*‘How the meetings go is often dependent on who is in charge of the meeting and if it’s clear who is supposed to be in charge…. That there’s someone who kind of steers things, that’s really important.’* KLLHP3

QUOTES SHOWING SIMILARITIES IN VERA’S, INGA’S AND BO’S SCENARIOS

**Beneficial with relational continuity in care**

*‘…you can follow up things and you know…you get to know each other and know how…when it’s a good time and how to do it and you can have individual planning for the patients.’* VLLHP1

*‘I think continuity is priceless…it means that I know my patients and if anything happens, you know that like, okay, this is no big deal, I don’t need to sound the alarm, because I know this person and know that we can wait. It is someone else, they might have to go in by ambulance, because they don’t know that patient. So that results in unnecessary care…’* SLLHP2

*‘But if it’s possible, I prefer to meet my patients again, because that makes things easier for me and it makes things easier for the patients. Because I know the patient and I know the patient has trouble understanding me…I know a bit about the patient’s background…can I explain everything by phone or will that maybe not work? Do I have to inform someone else, maybe. Having that kind of background information provides a huge advantage.’* KLLHP6

*‘…if you know each other and have a face for each person, that makes things much easier. Then you don’t have any problems. Like, okay, things went wrong, but we can…I mean, we…there’s no hard feelings anywhere, since we know each other. You have that flexibility at a smaller hospital.’* KLLHP1

**Beneficial to meet the same healthcare workers**

*‘My local doctor, I’ve been seeing them for thirty years…that contact couldn’t be better…if someone new comes in, they don’t know anything about me. The doctor I’ve seen for so many years knows about all my ailments.’* VLLP5

*‘So that…that’s who I know and my mother has seen them and everything. Those are the people I feel most confidence for.’* VLLN4

*‘So, in part that’s just a sense of security. I know that now I’ll see my doctor. He knows all about me…there are no surprises there.’* SLLP1

*‘But here, I feel that after all this time, it’s been many years, they know who I am and I know both the doctors and the staff. That makes things much easier…’* KLLN2

**Challenging to meet different healthcare workers**

*‘I think it’s not good to have several different doctors. One will say one thing and the other doctor will say something else and then…then you don’t know anything.’* VLLP4

*‘And there’s been a lot of staff that’s been replaced, and that creates a sense of uncertainty for the older people. And for us, the next of kin. It makes things much easier if you have someone who knows your story.’* VLLN2

*‘Yeah, you…you don’t really feel confidence for healthcare. That’s what I think is the worst part, that you don’t get to see the same people.’* KLLP5

**Beneficial to meet different healthcare workers**

*‘…but it can be good, every now and then… other people might have a different perspective sometimes, so seeing someone else too, yeah…’* KLLP1

*‘That he (the doctor) wanted me to come visit him at home…yeah, I don’t know, I guess I wanted a bit more distance than that… He would call and we had long conversations. He needed his patients.’* VLLP3

*‘…it might be that it’s good to come in with a new, fresh perspective, looking at the person too and seeing things that maybe you don’t because you live in…the same routine, in a way.’* VLLHP6

*‘…I feel that when we’ve had…this person for two weeks, then it’s kind of nice to get another one. When you have the same person all the time, maybe for years and years, then maybe you feel that you’re not really developing…you’re just doing the same thing every day, you’re not learning anything new.’* KLLHP5

**Lack of personnel hinders relational and management continuity**

*‘…that’s one thing you notice, the lack of continuity among staff, that’s often connected to a lack of familiarity with routines and work methods. And that, in turn, can lead to…various problems with collaboration. That you don’t know how you’re supposed to work.’* VLLHP9

*‘…my first spontaneous thought is the lack of staff, which means we don’t have any continuity. Because there are too few nurses and a lot of temps working.’* SLLHP1

*‘So even though it’s a good thought, to minimize the number of staff around a patient, it’s hard if there’s no staff that wants to work, it’s as simple as that.’* KLLHP5

**Prioritising relational continuity in key areas**

*‘The cancer patients, we try to get them to go to the same district nurse in as far as possible…and then we have an internal agreement not to switch out the district nurse.’* VLLHP4

*‘I feel like if you’re doing intimate things, like showering and that kind of stuff, then maybe it’s not that much fun to be naked in front of everyone. But if I know it’s someone I’ve met before, that can be enough…’* KLLHP5

**Ensuring time for follow-up visits**

*‘I want there to be follow up. I’m here for a reason and if you do a bunch of tests, I want to know…what’s happening. What actions will we be taking now…so continuity is really important.’* SLLP2

*‘So, that’s a clear benefit, that you get to follow up on things. Now, I use that a lot, when I’ve met with a patient and done tests, I’ll book a phone follow-up in 2–3 days. I’ll do that myself…’* VLLHP8

*‘I make sure to book them with me, that I get the follow-up and feedback myself, so that…I’ll write that in the medical record, that I’m the nurse responsible for the patient, so everyone knows that.’* SLLHP2

*‘…we want to take part in all the care planning meetings that we can, to get information and meet the patients.”’* KLLHP5

**Minimising the number of people involved**

*‘Yeah, you know of think that, especially the doctors who come in more seldom, but keep coming back… Then you book those patients first, their own patients, I mean…’* VLLHP1

*‘When you book a visit to a district nurse for changing a dressing, for instance, if you’ve gone to my colleague here seven times, well, then I’ll book that visit with her. You try to do that, in as far as possible.’* KLLHP1

**Taking the time to understand each patient's preferences and needs**

*‘…I think that this…really thinking that every patient is unique and has their own knowledge about themselves and it is their needs and expectations that we have to meet. We can’t always do what they want, but we need to be informed about how they want things and how…what we should say, how active they want to be themselves…’* VLLHP5

*‘…I mean, you connect to some people really quickly, but others you have to give it a few days before they open up… It’s important to get a sense of that…’* KLLHP5

MANAGEMENT CONTINUITY

**Challenging to collaborate between care organisations**

*‘I’ve been sent from the thorax department to X and then back again. That says it all, the connections between them are so poor…they were surprised I had a wound in my loin when I got up to X, so they haven’t communicated…so I think the collaboration works really badly.’* VLLP3

*‘The healthcare providers don’t have much contact with each other, I should say that right off the bat. And it feels really silly, because I know that she…sometimes, one week she got a call from hospital X and the next week a call from hospital Y. Because she gets care in both X and Y, and in Z as well, which is the healthcare centre that is closest to her.’* VLLN1

*‘The healthcare providers have very poor contact with each other, to put it in just a few words...’* SLLP4

*‘What my heart doctor had said, it…didn’t seem like the people at the lung department cared all that much about that…and I think that’s a really important part, that they can collaborate.’* KLLP4

*‘…you notice when the patients call us and are going to one doctor’s appointment one week and another the next week, or even the same week, then you can see that the healthcare providers don’t know about each other.’* VLLHP1

*‘…there are actually no clear routines on how we communicate… When we’re standing there on Friday afternoons and there’s no medication or anything and the patient is at home…who’s supposed to solve that? …that becomes stressful, really stressful.’* SLLHP2

*‘…but the hospital…I don’t feel like they really have any insight into how we work and so on… Because it feels like sometimes…the hospital doesn’t really have any idea…it feels like they’re there, doing their thing and what happens later, that’s the municipality’s problem and you’re done. That’s my perception!’* KLLHP2

**Time-consuming to collaborate and coordinate care**

*‘Every time he gets an appointment, I have to sit down and make a few calls to book a service trip for him. It takes a few calls, because usually you have to call the clinic he’s going to, or the place he’s going to, to confirm that he’s eligible for a trip, and then I have to call to book the trip, and then I have to call him and make sure he understands when the transport is coming. And preferably check that the transport really came.’* SLLN1

*‘…but the contacts that have to be made, when there are so many places involved, from advanced care at home to the municipality and physical therapy and other care instances…All that has to be done by phone and then it’s like…Sometimes you can’t even reach each other or…so I think that’s the hardest part of my job, actually.’* SLLHP9

*‘The nurses have a coordinator who usually takes the calls nowadays, and then maybe they’ve been to see my lady or guy and I want to know what they’ve done or just have questions. The coordinator doesn’t know exactly what’s been done…but then I feel like, well, I want to get in touch with them…yeah, because it becomes difficult, because if I don’t get an answer and then I have to keep trying...it takes a lot of time needlessly… In the past, you had their number and you called and talked to them personally.’* KLLHP5

**Defining responsibilities and regular communications**

*‘So, I have my areas connected to the home care groups…so they know that “right, I’m supposed to contact X”.’* VLLHP9

*‘…I think that when you run into an issue that you can resolve within your profession, and see that other types of efforts are needed, I feel that we are pretty good at seeing what area or responsibility falls into which category…’* VLLHP9

*‘Us local healthcare providers are in contact with the healthcare centre almost every week, at the management level and the nurse level, and almost daily, at the physical therapy level.’* VLLHP5

*‘We’ve set up a collaboration with healthcare centres and the hospital, where we sit in meetings for half an hour every day, to review the patients who are hospitalised…and where there’s a need for help from us…’* KLLHP2

**Care planning meetings**

*‘Well, of course they listened to me during care planning...’* VLLP3

*‘No, but the care planning was pretty good…I felt that I could speak for myself and as I recall, they fixed it quickly, so that I could go home.’* SLLP4

*‘The care planning was okay, they discussed what I needed, so that was no problem.’* KLLP3

*‘At the care planning meetings, it’s as much about getting it all out in the open and figuring out misunderstandings. It’s really good for everyone to sit together and get to hear about everything at the same time. And it clarifies roles. And provides information on who the patient should turn to.’* VLLHP7

*‘It was a really good care planning meeting and I think it helped both the patient and everyone else involved, having a clear plan about what to expect. And a more realistic time plan too, which was great.’* SLLHP6

*‘The idea is that we collaborate around the patient, and that’s what we do during this kind of meeting, so it’s really good that we all sit together like this, because then the social worker knows what we’ve planned. We know what the social worker has planned. We know what information the home care workers get… Yeah, it’s better when everyone hears the same information and has the chance to ask questions at that time. Instead of trying to find the information. And I feel it’s easier if you know who has decided what, so you can aim a specific question at the right person.’* KLLHP3

INFORMATIONAL CONTINUITY

**Having sufficient knowledge about health and care plans**

*‘…and I don’t have the energy to inform them about what others…like interventions. Often, I feel like you have, uhm…so little time, so it’s limited to what is…uhm…specific at the time, the reason you’re there for that particular visit…”* SLLP4

*‘Yeah, I tell them a lot when I’m there and what I’ve done and so on. I keep tabs on things and I look at the date in my bank and my tests and follow along in my medical records and, yeah…so I know…and if I feel that something is wrong, maybe I’ll call and ask about that, right.’* KLLP1

**Challenging to obtain information**

*‘Yeah, you’d really like to know more about how, how are things going. Should I be worried? Should I ignore this or should I be a bit concerned underneath it all?”* VLLP1

*‘Yeah, I mean, I’m so jaded when it comes to care interventions, but…you feel a bit powerless because…if you call, you don’t get anything anyway…very little information, because it’s all confidential…’* VLLN4

*‘…we get information about the interventions from the patient or their next of kin…because they usually inform us about…like what’s going on around them… At least when it comes to planning home care, for instance.’* VLLHP9

*‘…I don’t know a damn thing about what’s going to happen to me. I don’t get any information. And they just drag their feet.’* SLLP3

*‘Well, first, I think that feedback afterwards, that there could be some kind of…that you can review documentation or something showing what has happened…if you got that in writing or whatever, like, that this is what we talked about and this is what we decided together.’* SLLN1

*‘We don’t get that much information at all about physical therapy or inpatient care or…what we do find out is from the patient and sometimes home care or next of kin…other than that, we’re more or less on the outside looking in.”* SLLHP2

*‘But it’s hard, I feel, in some way, to have the planning we had. And I probably didn’t understand what they meant at the time…’* KLLP5

*‘But I think that there should be some kind of log…what’s been done? What happened? Up-to-date phone numbers, you can call this number or this number when you need to.’* KLLN2

*‘Ultimately, you can usually find the right information somewhere in all the papers, but then it’s usually easier to ask the patient, because they know right away. For me it can take ten, fifteen minutes before I find everything. Sometimes they’ll say: “But you can see that in the medical records.” Well, yes, it’s in there, but it’s hard to find.’* KLLHP6

**Relying on the patient’s medical records**

*‘The only knowledge I have about the patients is what I can read…I mean, from the medical records. I can go in and read about what’s happening down at Dermatology, for instance. I’ll go in and look at the medical records. Because I have to do that, since I want to know what they said down at that department and how we’re supposed to manage the patient.’* VLLHP4

*‘We have permission to read each other’s medical records…patient medical records and we’ll look at a patient’s medical records even if the person is hospitalised, so we take a lot of responsibility for the people who are hospitalised here…’* SLLHP3

*‘The information transfer and communication with other healthcare providers is a challenge. In X, where we work, we have several different healthcare providers with different medical record systems and we have the national patient overview system, but there is a lot of lag there, so we might not see everything and it can take a long time before important things that we need to know are included there.’* SLLHP4

*‘I can access the medical records and read about what going on. We have the national patient overview system as well, and we can access that and read it, if we have the patient’s consent. Then we can check, what was said at that doctor’s appointment and what was decided?’* KLLHP2

*‘We don’t have access to any medical records, we can’t see the notes of the occupational therapist or physical therapists or anything like that.’* KLLHP4

**Clear dialogue and direct communication**

*‘We have a pretty open climate, where you can like knock on a door and sit down and discuss things, so I think that works pretty well.’* VLLHP8

*‘I probably try to be very communicative…because there needs to be some clarity with a care plan, for instance, about who does what. I try to communicate that, whether it’s to the municipality, next of kin, or – of course – the patient. I want to have a dialogue, to ensure that we have understood each other.’* SLLHP9

*‘I really like have a more or less direct line to physical therapy, for instance, and since they don’t have access to our medical record system, it’s often the case that I have to…inform or enlighten them about a specific patient.’* SLLHP9

**A unified communication system**

*‘We have a system called Life Care. And that’s where we get a message about a person being hospitalised and given inpatient care, for instance, and that’s also where documentation takes place. It’s really intended for the individual patient. So, it’s through this tool that we can see a bit about the planning from other professions. The idea is that the person discharged from the department will get these documents printed out.’* VLLHP9

*‘I log in to the system Web-care when I work as a coordinator, to ensure that I don’t overlook anything they’ve written or asked. They usually ask, like, “I see that she or he is hospitalised, according to some district nurse and I’d like to be informed.” So, then I can write a note to calm them down, so they know that it isn’t time for the patient to go home yet.’* SLLHP1

*‘Those who are linked (into a unified communication system) get really well taken care of from day one, from the first hour when they come to the hospital, we know who they are and the hospital gets information on the care…what we know about the patient, some don’t have home care or personal alarms, whereas others have the full spectrum, so to speak. Everyone writes what interventions they have going for each patient. It makes a huge difference that we are familiar with them, that we know all about them.’* KLLHP1
